# In Vitro and in Vivo Antiviral Activity of Mizoribine Against Foot-And-Mouth Disease Virus

**DOI:** 10.3390/molecules24091723

**Published:** 2019-05-03

**Authors:** Shi-Fang Li, Mei-Jiao Gong, Yue-Feng Sun, Jun-Jun Shao, Yong-Guang Zhang, Hui-Yun Chang

**Affiliations:** 1 State Key Laboratory of Veterinary Etiological Biology, OIE/National Foot-and-Mouth Disease Reference Laboratory, Lanzhou Veterinary Research Institute, Chinese Academy of Agricultural Sciences, Lanzhou 730046, Gansu, China; fruceslee@foxmail.com (S.-F.L.); jiaomgong@126.com (M.-J.G.); sunyuefeng@caas.cn (Y.-F.S.); shaojunjun@caas.cn (J.-J.S.); 2 Jiangsu Co-innovation Center for Prevention and Control of Important Animal Infectious Diseases and Zoonoses, Yangzhou 225009, Jiangsu, China

**Keywords:** foot-and-mouth disease virus, foot-and-mouth disease, mizoribine, antiviral, in vivo

## Abstract

Foot-and-mouth disease (FMD) is a highly contagious viral disease of cloven-hoofed animals, which has significant economic consequences in affected countries. As the currently available vaccines against FMD provide no protection until 4–7 days post-vaccination, the only alternative method to control the spread of FMD virus (FMDV) during outbreaks is the application of antiviral agents. Hence, it is important to identify effective antiviral agents against FMDV infection. In this study, we found that mizoribine has potent antiviral activity against FMDV replication in IBRS-2 cells. A time-of-drug-addition assay demonstrated that mizoribine functions at the early stage of replication. Moreover, mizoribine also showed antiviral effect on FMDV in vivo. In summary, these results revealed that mizoribine could be a potential antiviral drug against FMDV.

## 1. Introduction

Foot-and-mouth disease (FMD) is one of the most economically and socially devastating diseases affecting cloven-hoofed animals [1]. The infectious agent, foot-and-mouth disease virus (FMDV), is a member of the *Aphthovirus* genus of the *Picornaviridae* family, and contains single-stranded positive-sense RNA genomes of about 8,500 nucleotides [2]. As an antigenically variable virus, FMDV consists of seven serotypes (A, O, C, Asia 1, and South African Territories 1, 2, and 3) and a large number of subtypes. In general, slaughtering FMDV-infected/exposed or FMDV-susceptible animals, restricting animal movement, and, in some cases, vaccinating against FMDV and then slaughtering these animals are used as control measures for potential outbreaks in disease-free areas [3]. Although inactivated FMD vaccines have been available since the early 1900s and new novel vaccines are being continuously developed, they offer little or no cross-protection against various serotypes and subtypes of FMDV. In addition, these vaccines do not provide complete clinical protection until seven days post-vaccination. Therefore, there is a need for developing effective and safe alternative antiviral strategies against FMDV [4,5,6].

**Figure 1:**
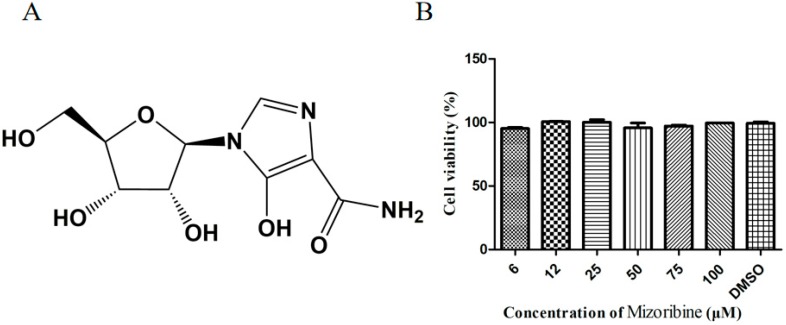
The cytotoxic effect of mizoribine treatment on IBRS-2 cells. (**A**)The chemical structure of mizoribine. (**B**) The cytotoxic effect of mizoribine. The IBRS-2 cells were treated with 6, 12, 25, 50, 75, and 100 μM mizoribine for 72 h. Relative cell viability was determined by MTS assay and normalized to the value of 1% DMSO-treated group (set at 100 %). Data are expressed as the mean ± SD of three independent experiments.

Mizoribine, an imidazole nucleoside (Figure 1A) [7], has been used as an immunosuppressive agent for the treatment of renal transplantation, autoimmune diseases, and steroid-resistant nephrotic syndrome in some countries owing to its antiproliferative activity against T and B lymphocytes [7]. This drug could be phosphorylated by adenosine kinase and converted to mizoribine 5′-monophosphate, the active form of mizoribine. It has been demonstrated that mizoribine 5′-monophosphate acts as an inhibitor of inosine 5′-monophosphate dehydrogenase (IMPDH) and guanosine monophosphate synthetase [8]. In addition, mizoribine is known to inhibit replication of some DNA and RNA viruses, such as cytomegalovirus [9], respiratory syncytial virus [10], severe acute respiratory syndrome-associated coronavirus (SARS-CoV) [11], bovine viral diarrhea virus (BVDV) [12], vaccinia virus [13], influenza virus types A and B, and herpesviruses, in combination with acyclovir [14,15]. However, the antiviral activity of mizoribine against FMDV has not yet been investigated. Hence, in this study, the antiviral effect of mizoribine against FMDV was evaluated in vitro using IBRS-2 cells and confirmed in vivo using suckling mice.

## 2. Results

### 2.1. Cytotoxicity of Mizoribine on IBRS-2 Cells

Figure 1B illustrates the results of the MTS assay. As is shown in Figure 1B, mizoribine presented little or no cytotoxicity to the cells. The cell viability was 95.14%, 100.74%, 100.19%, 95.71%, 97.22%, and 99.51% at mizoribine concentrations of 6, 12, 25, 50, 75, and 100 μM, respectively, and the 50% cytotoxic concentration (CC_50_) of mizoribine was estimated to be more than 100 μM on IBRS-2 cells.

### 2.2. Antiviral Effect of Mizoribine on FMDV Replication in IBRS-2 Cells

**Figure 2:**
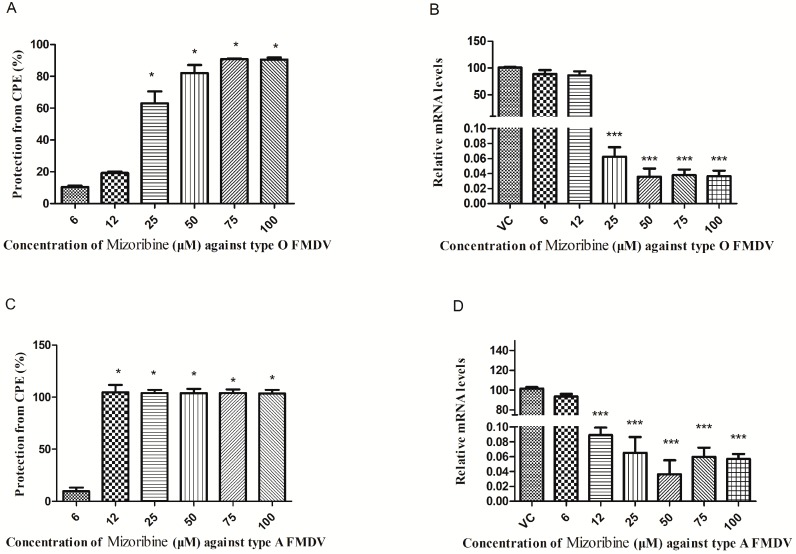
Anti-foot-and-mouth disease (FMD) virus activity of mizoribine in IBRS-2 cells. Confluent IBRS-2 cells infected with 100 TCID_50_ (**A**,**B**) O/MY98/BY/2010 and (**C**,**D**) A/GD/MM/2013, were exposed to different concentrations of mizoribine for 48 hr. “VC” (virus control group) represents those cells treated with 1% DMSO without mizoribine. (**A**,**C**) Cell viability was measured using MTS assay. Results are expressed as the mean ± SD from three experiments. (**B**,**D**) The supernatants were used for viral RNA quantification using qPCR. The expression values relative to that of β-actin were calculated using the 2^−ΔΔCt^ method. CPE, cytopathic effect. Statistically significant differences are indicated by asterisks (* *p* < 0.05, *** *p* < 0.001).

The inhibitory effect of mizoribine on FMDV infection in IBRS-2 cells was calculated by measuring cell viability using the results of MTS assay. As indicated in Figure 2A, the inhibition rates were approximately 63.01%, 82.03%, 90.89%, and 90.41% in cells treated with 25, 50, 75, and 100 μM mizoribine, respectively, whereas other lower mizoribine concentrations demonstrated limited or no inhibitory effect on FMDV. The IC_50_ and SI values were calculated to be 21.39 μM and 4.67, respectively. Interestingly, mizoribine also displayed activity against another FMDV strain, A/GD/MM/2013, with an IC_50_ of 6.57 μM and SI value of 15.20 (Figure 2C). These data supported the potential broad-spectrum activity of mizoribine against RNA viruses.

**Figure 3:**
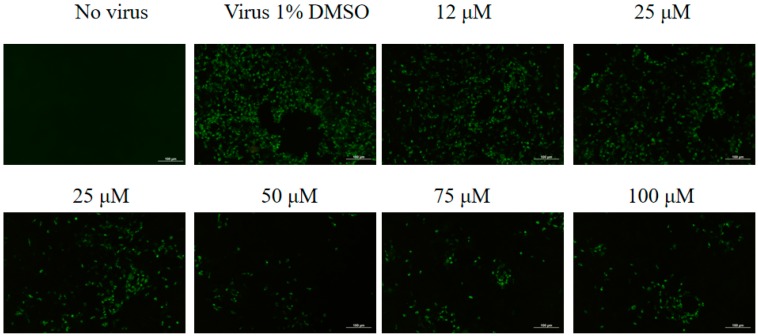
Anti-foot-and-mouth disease virus (FMDV) activity of mizoribine detected by immunofluorescence microscopy. IBRS-2 cells were infected by 100 TCID_50_ O/MY98/BY/2010 with or without treatment by increasing concentrations of mizoribine for 12 h. The whole viral proteins were determined by IFA, the green fluorescence represents the intracelluar distribution of FMDV. Scale bars indicate 100 μm.

The transcription of FMDV mRNA was significantly reduced with 25, 50, 75, and 100 μM mizoribine treatments relative to the DMSO control group (Figure 2B). Consistent with mRNA expression, immunofluorescence assay (IFA) revealed that mizoribine inhibited the expression of FMDV protein in a dose-dependent manner (Figure 3). The concentration of 75 μM, which showed more protection against CPE than other concentration, was chosen for the further study.

**Figure 4:**
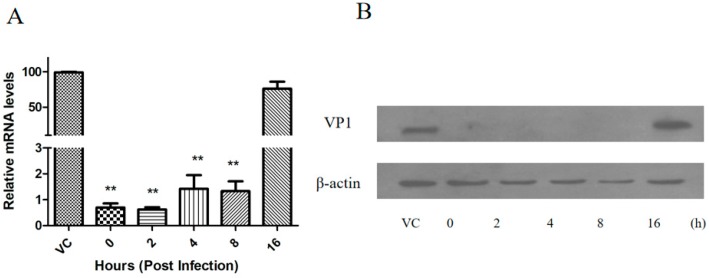
Time points of drug-addition study in FMDV replication. IBRS-2 cells were infected with 100 TCID_50_ O/MY98/BY/2010 followed by treatment of 75 μM mizoribine at indicated time (hours post-infection (hpi)). Virus levels were determined at 48 hpi by (**A**) Q-PCR and (**B**) Western blot. “VC” represents those cells treated with 1% DMSO without mizoribine. Values represent the mean ± standard deviation for three independent experiments. The asterisks indicate significant differences between mock-treated and drug-treated cells (** *p* < 0.01).

Subsequently, the antiviral efficacy of mizoribine was further evaluated at various intervals post-FMDV infection, we found that the viral 2B mRNA and protein expressions were continuously inhibited at different time points (0, 2, 4, and 8 h) after treatment with 75 μM mizoribine; however, no significant differences were observed between 16 h post-infection (hpi) and the control group (Figure 4). Taken together, these results suggested that mizoribine exhibited potent antiviral activity against FMDV in IBRS-2 cells at the early stages of viral infection.

### 2.3. Effect of Mizoribine on Purine Synthesis in FMDV

**Figure 5:**
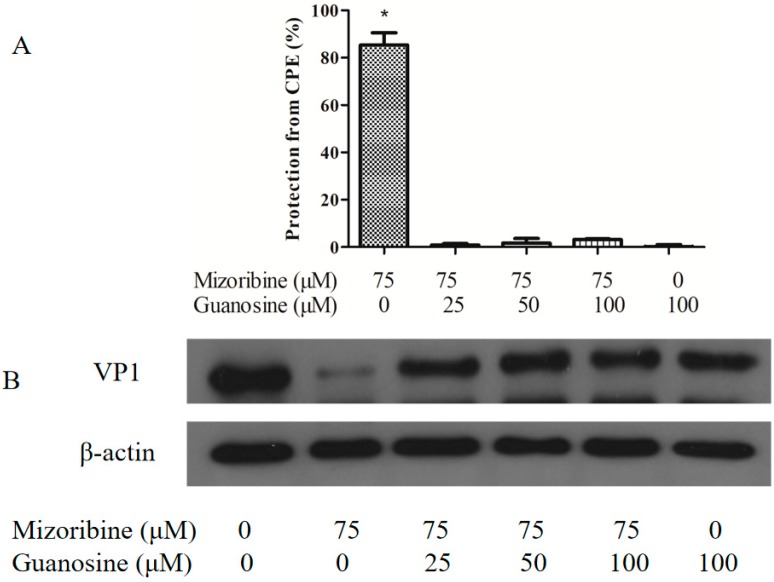
Guanosine supplementation attenuated the anti-FMDV effect of mizoribine. Serial dilutions of guanosine (from 100 to 25 μM) were added to FMDV (100 TCID_50_ O/MY98/BY/2010)-infected IBRS-2 cells, when treated with 75 μM mizoribine for 48 h. (**A**) The cell viability of cells and (**B**) FMDV VP1 levels were analyzed as described above. Data are the mean ± SD of three independent experiments. * *p* < 0.05.

Inosine-5′-monophosphate dehydrogenase (IMPDH) is required for de novo purine nucleotide synthesis and its inhibition can lead to depletion of intracellular GTP pools. To investigate the effect of mizoribine on purine synthesis in FMDV, serially diluted guanosine was added to the infected cells treated with mizoribine. While guanosine had no effect on mizoribine, it significantly attenuated the anti-FMDV effect of mizoribine in a dose-dependent manner (Figure 5). These data indicated that mizoribine activity against FMDV involved inhibition of IMPDH-dependent purine synthesis.

### 2.4. Antiviral Activity of Mizoribine in Vivo

**Figure 6:**
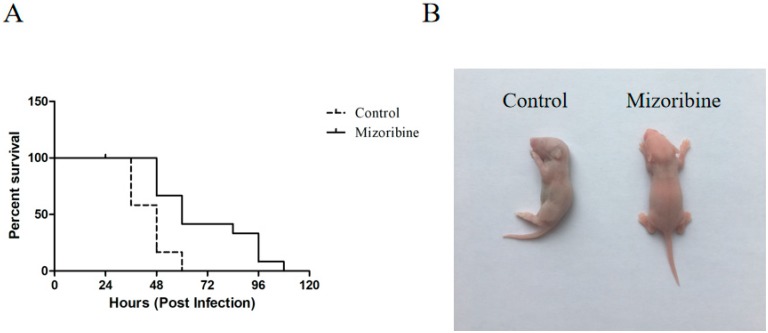
Mizoribine treatment prolonged the survival time of FMDV-infected mice. (**A**) Survival rates of the FMDV-infected mice treated with the placebo or mizoribine (50 μg) were recorded at five days (N = 12). (**B**) Morphological observation of the suckling mice after FMDV infection at 60 hpi.

The suckling mice pretreated by subcutaneous injection of mizoribine or PBS in the neck were infected with FMDV to determine the antiviral activity of mizoribine in vivo. All the solvent-treated mice died within 60 h after 100 LD_50_ of O/MY98/BY/2010 challenge. In contrast, a 48-h delay in death post-infection was noted in the mizoribine-treated group, and all the mice died within 108 h after the viral challenge. The overall death time of mice treated with mizoribine was delayed by 48 h, when compared with the control, and a significant difference in mouse survival was noted between the treatment group and control (*p* = 0.0014) (Figure 6A).

**Figure 7:**
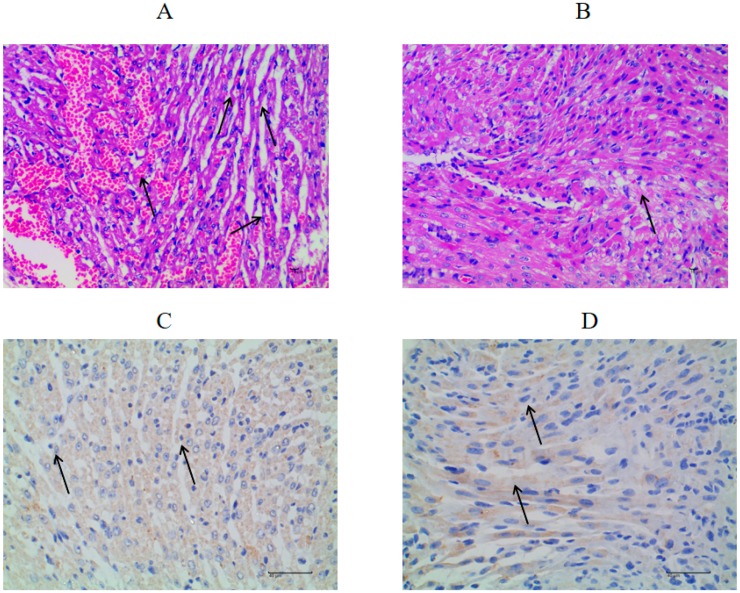
Histological and IHC analysis of heart tissues in FMDV-challenged suckling mice treated with mizoribine at 34 hpi. (**A**,**B**) The pathological changes of heart tissues were observed after H&E staining. Mice were sacrificed at 34 h post-challenge. Heart tissue were then collected and fixed in 4% paraformaldehyde solution, paraffin embedded, sectioned, and stained with H&E. Representative images from (**A**) control group and (**B**) treated group are shown (N = 4). Tissue damage was identified and is indicated by black arrows (magnification, 400×). (**C**,**D**) The immunohistochemical expression of FMDV for heart tissue was detected using IHC. Representative images from (**C**) control group and (**D**) treated group are shown (N = 4). FMDV antigen was identified and is indicated by black arrows (magnification, 400×).

Furthermore, significant histopathological damage was observed in the heart tissue of FMDV-infected mice, including considerable myocardial interstitial hemorrhage, myocardial fibronectin degeneration, and extensive inflammatory cell infiltration, as indicated by black arrows (Figure 7A). In addition, myocardial fiber edema and incomplete fibrous structure were also observed. However, mice treated with mizoribine showed mild histopathological changes and only a small amount of inflammatory cell infiltration in the heart (Figure 7B). Intriguingly, although FMDV antigen was detected in the heart tissue of both mizoribine-treated and control mice, it was not statistically significant (Figure 7C,D). These findings suggested that histopathological damage may be the main cause of death resulting from FMDV infection, and mizoribine can effectively alleviate these effects.

## 3. Discussion

As FMDV exhibits high mutation rates and produces significant economic loss in affected countries, it is important to adopt effective measures to control this virus. It has been demonstrated that mizoribine does not produce tumorigenic and gonadal suppression effects, and exerts low bone marrow inhibition and hepatotoxic outcomes, and has been used for the treatment of renal diseases in humans [8,16]. Thus, considering the safe, reliable, and acceptable efficacy of mizoribine in humans, it might also be employed for the treatment of animal diseases.

To the best of our knowledge, the present study is the first to report on the antiviral effect of mizoribine on FMDV both in vitro and in vivo. By using MTS assay, the cytotoxicity of mizoribine was determined to be very weak, with a CC_50_ value higher than 100 μM. Furthermore, mizoribine showed significant anti-FMDV activity, not only against type O FMDV O/MY98/BY/2010 strain, but also against type A FMDV A/GD/MM/2013, with SI values of 4.67 and 15.20, respectively. These results indicated that mizoribine could be a better drug to prevent FMDV A/GD/MM/2013 infection. Moreover, qPCR and IFA findings revealed that mizoribine can significantly inhibit the viral mRNA and FMDV protein levels. To understand the preliminary antiviral mechanism of mizoribine, time-of-drug-addition assay was performed, and the results demonstrated that mizoribine mainly functions at the early stages of infection. It has been reported that the antiviral activity of mizoribine involves inhibition of IMPDH [7], an essential enzyme for the synthesis of guanosine monophosphate from inosine monophosphate through de novo pathway [8,17]. Similarly, in the present study, the antiviral activity of mizoribine against FMDV was found to be attenuated by guanosine supplementation. The animal experiment demonstrated that mizoribine had an inhibitory effect on FMDV in vivo, and treatment with 50 μg of mizoribine significantly prolonged the survival of FMDV-infected suckling mice. Although the in vivo results did not show a significant increase in the survival rates of infected mice, the delay in death and alleviated histopathological changes suggested the inhibitory effect of mizoribine on viral replication. Overall, the weak cytotoxicity and strong antiviral activities of mizoribine both in vitro and in vivo favored its further potential clinical applications in the treatment of viral infection.

Combination treatment strategies have been proposed to enhance the efficacy of antiviral agents, because of their advantages in overcoming viral mechanisms of resistance to antiviral treatments [18]. It has been demonstrated that mizoribine enhances the anti-caprine-herpesvirus-1 activity of acyclovir, and the combination of mizoribine and acyclovir resulted in an almost complete inhibition of viral replication. Thus, combined therapy of acyclovir and mizoribine could be exploited for the treatment of genital infection by herpesviruses [19]. Moreover, mizoribine has been reported to be active against the replication of BVDV in Madin-Darby bovine kidney (MDBK) cells, and a combination of interferons (IFNs) and mizoribine had been noted to synergistically inhibit BVDV replication in bovine kidney cells [17]. With regard to FMD, FMDV have been demonstrated to be very sensitive to IFNs, and IFN-based strategies have been established to be an efficient biotherapeutic option against FMDV [20]. Similarly, the combination of antiviral agents, such as siRNA, ribavirin, and IFNs, has been determined to produce enhanced antiviral effect against FMDV [18]. Therefore, future studies must investigate whether a combination of IFNs and mizoribine could produce increased inhibitory effect on FMDV replication both in vitro and in vivo.

In conclusion, to the best of the authors’ knowledge, the present study is the first to demonstrate that mizoribine can suppress FMDV replication in vitro as well as prolong the survival of suckling mice in vivo, suggesting the potential applications of this drug in antiviral regimens for FMD treatment. The findings of this study may warrant further investigations on the efficacy and safety of combined use of mizoribine and other antiviral agents in vitro and in vivo.

## 4. Materials and Methods

### 4.1. Materials

IBRS-2 (swine kidney cell line) cells were preserved in our laboratory and cultured in complete Dulbecco’s modified Eagle’s medium (DMEM) containing 10% fetal bovine serum (Gibco, Grand Island, NY, USA) and supplemented with penicillin and streptomycin (10 units/mL, Gibco). The cells were maintained in 5% CO_2_ at 37 °C. Mizoribine and guanosine were purchased from MCE (MedChemExpress, New Jersey, NJ, USA), dissolved in dimethyl sulfoxide (DMSO) and stored at −20 °C. A SPlink detection kit and peroxidase-conjugated goat anti-rabbit IgG (H + L) were purchased from ZSGB (Beijing, China). The anti-β-actin mouse polyclonal antibody was purchased from Sigma-Aldrich (Sigma-Aldrich, St Louis, MO, USA). Horseradish peroxidase (HRP)-conjugated goat anti-mouse IgG or anti-rabbit IgG were purchased from ZSGB (Beijing, China). Type O FMDV VP1 rabbit polyclonal antibodies were kindly provided by Haixue Zheng (OIE/National Foot-and-Mouth Disease Reference Laboratory). Hyperimmune serum of FMDV (O/MYA98/BY/2010) was prepared in our laboratory. A PrimeScript™ RT reagent kit containing gDNA Eraser and SYBR Premix Ex Taq^TM^ II (Tli RNaseH Plus) was purchased from TaKaRa (Dalian, China). MTS assay were available from Abcam (Cambridge, UK). FMDVs (O/MYA98/BY/2010 and A/GDMM/CHA/2013) were used to investigate the antiviral activity of mizoribine. The 50% tissue culture infective dose (TCID_50_) was measured with the Reed and Muench method.

### 4.2. Laboratory Animals

Thirty-two (32) two- and three-day-old BALB/c mice weighing 3–4 g were used to investigate the efficacy of mizoribine in vivo. All the animal trials were performed in a Biosafety level-3 laboratory and approved by the Animal Ethics Committee of Lanzhou Veterinary Research Institute, Chinese Academy of Agricultural Science (No. LVRIAEC2018-007). FMDV type O (O/MYA98/BY/2010) strain was used for viral challenge.

### 4.3. Cytotoxicity Assay

The cytotoxicity of mizoribine was evaluated using MTS assay. Briefly, 3 × 10^4^ cells were seeded into each well of a 96-well plate containing 100 μL of complete medium. On the following day, the cells were incubated with 100 μL of mizoribine at various concentrations (6, 12, 25, 50, 75, and 100 μM) for 72 h. As a control, cells were treated with 1% DMSO. After treatment, the supernatants were discarded, and the cells were washed three times. Then, 100 μL of DMEM were added to each well along with 20 μL of MTS solution and incubated for an additional 3 h at 37 °C. The optical density of each well at 490 nm was determined using a microplate reader (Bio-Rad, Hercules, CA, USA). The cell viability was expressed as the percentage of absorbance of the treated cells to that of the control cells.

### 4.4. Antiviral Assay

The antiviral activity of mizoribine against FMDV was determined using an MTS-based cytopathic effect (CPE) inhibition assay. Briefly, the viral suspension (100 TCID_50_ O/MY98/BY/2010) was added to the IBRS-2 cell monolayers. After incubation for 1 h, the cells were washed three times with DMEM, and serially diluted mizoribine solution was added to the wells (eight wells for each concentration). Then, the plates were incubated at 37 °C in 5% CO_2_. After 48 h, when maximum CPE was noted in the virus control group (VC), the cell viability was measured using MTS assay as described earlier, and the percentage of inhibition associated with each mizoribine concentration was normalized with respect to the VC. The virus inhibition rate was calculated as follows: inhibition rate = (optical density of mizoribine group − optical density of VC)/(optical density of cell control group − optical density of VC) × 100%. The supernatant of each well was collected and the viral 2B mRNA was analyzed using Q-PCR. The 50% inhibitory concentration (IC_50_) values were calculated with GraphPad software (version 7.04, La Jolla, CA, USA).

### 4.5. Guanosine Supplementation Experiment

The monolayers of cells were seeded in 96-well plates infected with 100 TCID_50_ FMDV O/MY98/BY/2010 at 37 °C for 1 h. Then, a 75 μM portion of mizoribine supplemented with serial dilutions of guanosine (from 100 to 25 μM) was added and incubated for 48 h. After incubation, the viral protein and gene expressions were assessed by Western blot analysis and Q-PCR, respectively.

### 4.6. Time-of-Drug-Addition Assay

The IBRS-2 cells were incubated with 100 TCID_50_ O/MY98/BY/2010 for 1 h. Subsequently, the viruses were removed and the medium was replaced. Mizoribine (75 μM) was added to the cells during infection (co) or post-infection (2, 4, 8 and 16 h). After 48 h incubation, the FMDV 2B mRNA and VP1 protein in the cells were determined by Q-PCR and Western blot analysis, respectively.

### 4.7. Q-PCR

The expression levels of FMDV 2B mRNA and β-actin were determined by real-time PCR as previously reported [21]. Briefly, the total RNA from the IBRS-2 cells was extracted using TRizol reagent, and 1 μg of RNA was used in reverse transcription reaction using a PrimeScript™ RT reagent kit containing gDNA Eraser, following the manufacturer’s instructions. The reaction mixture for real-time PCR comprised diluted cDNA (1 μL), 10 μM primers, and 12.5 μL of SYBR Green Master Mix to a final volume of 25 μL. The amplification conditions were as follows: 95 °C for 30 s, followed by 40 cycles of 95 °C for 5 s, 56 °C for 30 s, and 72 °C for 30 s. Dissociation curves were generated to analyze the individual PCR products after 40 cycles. The expression levels of FMDV mRNA genes were normalized against those of porcine β-actin mRNA. The analyses of the relative gene expression data were performed using the 2^−ΔΔCT^ method [22].

### 4.8. Western Blot Analysis

For Western blot analysis, the cells were lysed with Pierce RIPA, and the cell lysates were resolved and separated by 12% SDS-PAGE and transferred to polyvinylidene fluoride membrane. The membranes were probed with primary antibodies against FMDV VP1 protein (dilution, 1:1000) to detect virus replication. A monoclonal antibody to β-actin (dilution, 1:4000) was used as a loading control. The membranes were incubated with goat anti-mouse and anti-rabbit conjugated with horseradish peroxidase for 1 h at room temperature and examined by Pierce™ ECL Western Blotting Substrate.

### 4.9. Indirect Immunofluorescence Assay

To determine the antiviral activity of mizoribine, indirect immunofluorescence assay (IFA) was performed as previously described with minor modifications [23]. The IBRS-2 cells were seeded into a 12-well plate at a concentration of 3 × 10^5^ cells/well and incubated for one day. Subsequently, the cells were incubated with 100 TCID_50_ FMDV for 1 h, and mizoribine diluted at indicated concentrations was added to the cells after removal of the viral inoculum. After 12 h of incubation, the IBRS-2 cells were washed thrice with PBS and fixed with 4% paraformaldehyde for 10 min. Then, paraformaldehyde was removed and absolute methanol was added to the cells and incubated for 5 min. Subsequently, the cells were washed with PBS and blocked using blocking buffer (PBS supplemented with 0.3% Triton X-100 and 10% FBS). Rabbit hyperimmune serum raised against type O FMDV (O/MY98/BY/2010) was used to probe type O FMDV, and peroxidase-conjugated goat anti-rabbit IgG (H + L) was employed as secondary antibody. Finally, the cells were counterstained with 4′, 6-diamidino-2-phenylindole (DAPI) and viewed under fluorescence microscope (Nikon ECLIPSE TS100 fluorescence microscope, Yokagawa Electric Corporation, Tokyo, Japan).

### 4.10. Antiviral Activity of Mizoribine in Vivo

Specific-pathogen-free three-day-old Kunming suckling mice were inoculated with 50 μg of mizoribine dissolved in 10 μM DMSO, 5% Tween 80, and 0.1 mL of PBS by subcutaneous neck injection. The negative control was inoculated with PBS. After 2 h, viral challenge was performed by intradermal injection with 100 50% lethal dose (LD_50_) FMDV serotype O O/MY98/BY/2010 into the subcutaneous neck region of the mice. The animals were monitored for five days, and a log-rank test was performed for statistical analysis using GraphPad software. At 34 h post-infection, the suckling mice were euthanized and processed for histological and immunohistochemical investigations.

For histopathological analysis, the heart tissues were fixed in 4% paraformaldehyde solution, embedded in paraffin, and cut into 4 μm thick sections for standard hematoxylin and eosin (H & E) staining. With regard to immunohistochemical (IHC) studies, the FMDV antigen was detected as follows: 5 μm thick paraffin-embedded tissue sections were deparaffinized and treated with methanol-hydrogen peroxide for 10 min before heat-induced antigen retrieval in 0.01 M sodium citrate buffer (pH = 6) for 30 min. Rabbit hyperimmune serum raised against type O FMDV (O/MY98/BY/2010) was used as primary antibody. The tissue sections were processed with a SPlink detection kit for 30 min and stained with DAB for 2 min at room temperature. After washing, the tissue sections were counterstained, mounted, examined under a digital microscope (BA400Digital, Motic, Xiamen, China), and photographed. The optical density of each tissue section was determined by Image-Pro Plus 6.0 software (Media Cybernetics, Rockville, MD, USA).

### 4.11. Statistical Analysis

All the data are expressed as mean ± standard deviation (SD) for at least three independent experiments. One-way ANOVA was used to analyze the difference between mizoribine and control groups using GraphPad Prism 7 (GraphPad Software, Inc., La Jolla, CA, USA), version 7.04, and significant differences were defined at *p* < 0.05. Selective index (SI) = CC_50_/EC_50_.
